# Causal association between psoriasis vulgaris and bullous pemphigoid: a two-sample bidirectional Mendelian randomization study

**DOI:** 10.3389/fimmu.2024.1365118

**Published:** 2024-03-13

**Authors:** Aobei Zhang, Zhihui Yang, Tao Huang, Mingyue Wang

**Affiliations:** ^1^ Department of Dermatology and Venerology, Peking University First Hospital, Beijing, China; ^2^ Beijing Key Laboratory of Molecular Diagnosis of Dermatoses, Peking University First Hospital, Beijing, China; ^3^ National Clinical Research Center for Skin and Immune Diseases, Beijing, China; ^4^ National Medical Products Administration (NMPA) Key Laboratory for Quality Control and Evaluation of Cosmetics, Peking University First Hospital, Beijing, China; ^5^ Department of Epidemiology and Biostatistics, School of Public Health, Peking University, Beijing, China; ^6^ Key Laboratory of Molecular Cardiovascular Sciences (Peking University), Ministry of Education, Beijing, China; ^7^ Center for Intelligent Public Health, Academy for Artificial Intelligence, Peking University, Beijing, China

**Keywords:** Mendelian randomization, psoriasis vulgaris, bullous pemphigoid, causal, genetic

## Abstract

**Background:**

The association between psoriasis vulgaris and bullous pemphigoid (BP) remains largely unknown.

**Objectives:**

To investigate whether there is a causal effect between psoriasis vulgaris and BP.

**Methods:**

Two-sample bidirectional Mendelian randomization (MR) analyses were conducted using publicly released genome-wide association studies (GWAS) summary statistics. The GWAS summary statistics for BP were downloaded online from FinnGen Biobank Documentation of the R12 release, which includes 219 BP cases and 218,066 controls. The GWAS data for psoriasis vulgaris were extracted from Sakaue et al., which comprises 5072 cases and 478,102 controls. Single-nucleotide polymorphisms (SNPs) associated with exposure were selected as instrumental variables by performing additional quality control steps. The inverse-variance-weighted (IVW) method was used for the primary MR analyses, and the MR-Egger regression, weighted mode method, weighted median method, and simple mode were employed for sensitivity analyses. The MR-Egger intercept test and “leave-one-out” sensitivity analysis were performed to evaluate the horizontal pleiotropy and the potentially influential SNPs, respectively.

**Results:**

Genetically determined log odds of psoriasis vulgaris were associated with an increased risk of BP (IVW: odds ratio (OR) = 1.263, 95% confidence interval (CI): 1.013-1.575, *P*=0.038). Sensitivity analyses by the weighted mode (OR=1.255, 95%CI: 0.973-1.618, *P*=0.106), MR Egger (OR=1.315, 95%CI: 0.951-1.817, *P*=0.126), simple mode (OR=1.414, 95%CI: 0.823-2.429, *P*=0.234) and weighted median method (OR=1.177, 95%CI: 0.889-1.559, *P*=0.254) derived directionally consistent relationship between the genetically predicted log odds of psoriasis vulgaris and risks of developing BP. On the contrary, we found that genetically predicted BP had no significant effect on psoriasis vulgaris (IVW: OR=0.996, *P*= 0.707), indicating the unidirectionality of the relationship. MR-Egger intercept tests showed no evidence of horizontal pleiotropy. No influential SNP driving the results was detected by the leave-one-out sensitivity analysis.

**Conclusions:**

Our results suggested that psoriasis vulgaris causally increases the risk of BP, highlighting the need for potential strategies for the prevention and early diagnosis of comorbid BP in patients with psoriasis vulgaris. Further researches into this association and underlying mechanisms are warranted.

## 1 Introduction

Psoriasis is a chronic, immune-mediated, inflammatory skin disease characterized by the rapid proliferation of skin cells, resulting in the formation of distinct red scaly patches on the skin. Psoriasis vulgaris is the most common clinical variant (1, 2). Previous researches have suggested that autoreactive T cells contribute to the pathogenesis of psoriasis, which indicates that psoriasis may be autoimmune in nature (3–6). Additionally, psoriasis patients have been found to have a higher prevalence of various immune-mediated disorders, including autoimmune bullous diseases. (AIBDs) (7–9).

Bullous pemphigoid (BP) is a chronic autoimmune subepidermal blistering disease characterized clinically by urticarial pruritus and tense blisters on an erythematous background (10). It is associated with autoantibodies which directed against two hemidesmosomal proteins: BP180 (BP antigen2, collagen XVII) and BP230 (BP antigen 1) in the basement membrane zone of the epidermis (11). The specific mechanisms by which BP can be induced are unknown, although the majority are linked to factors such as genetics, drug intake, viral infections, physical agents, and diet that may locally disrupt the skin basement membrane zone (BMZ) (12, 13). In recent years, increasing researches have indicated a link between psoriasis and BP (14–16), especially psoriasis vulgaris (8). However, since potential confounding factors cannot be fully evaluated or controlled in observational studies, the causality of the link between psoriasis vulgaris and BP remains unknown. In order to have a deeper understanding of their pathogenesis and timely identify the patients at risk, it is crucial to evaluate the causal relationships between psoriasis vulgaris and BP.

Mendelian randomization (MR) is a typical method of drawing conclusions about causal relationships between exposures and outcomes by using genetic variations [i.e. single nucleotide polymorphisms (SNPs)] as instrumental variables (IVs) (17, 18). Since the genetic variations have formed before born, MR estimates are not affected by reverse causation or confounding factors, overcoming the limitations of observational investigations (19). In order to examine the causal relationship between psoriasis vulgaris and BP, a two-sample bidirectional MR analysis was carried out using publicly available summary statistics from genome-wide association studies (GWAS) data.

## 2 Materials and methods

### 2.1 Study design

**Figure 1:**
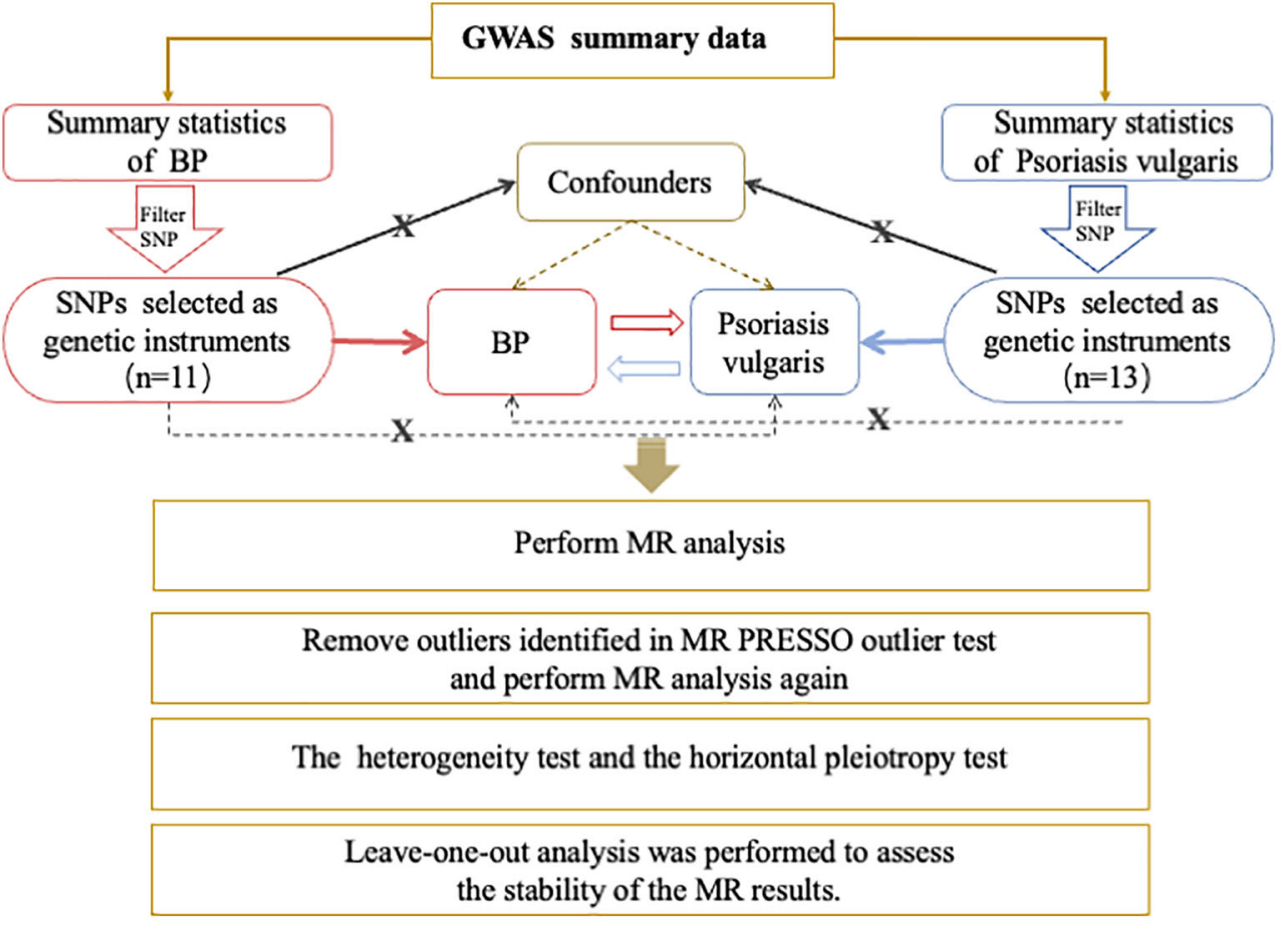
Overview of the Procedures in this bidirectional Mendelian randomization (MR) study. Brief description of the study design. For Psoriasis vulgaris, we filtered SNPs that met the genome-wide significance criteria of P<5e-08; while a lower threshold of P<5e-06 for BP. Then SNPs were pruned for independence using a clumping procedure with a linkage disequilibrium (LD) threshold of R2 > 0.001 in a 10,000 kb window and SNPs with *F*-statistic <10 were additionally excluded. The red and blue solid lines indicate that the selected SNPs were significantly associated with the exposure/outcome. The black solid lines indicate that the selected SNPs were not related to any confounders of the exposure–outcome association. The black dotted lines indicate that the selected SNPs exerted effects on the outcome only via the exposure. GWAS, genome-wide association study; BP, bullous pemphigoid; SNP, single nucleotide polymorphism; MR, Mendelian randomization;.

This was a bidirectional two-sample MR study. We used open-access GWAS data sets at https://gwas.mrcieu.ac.uk/to conduct all analyses. Genetic variants significantly associated with psoriasis vulgaris were used as IVs to estimate the causal effects of psoriasis on BP. Then we attempted to investigate the causality between BP and psoriasis vulgaris in the reverse direction. **Figure 1** demonstrates the study design, MR analysis procedure, and the three primary assumptions MR requires (19), which contains 1) a strong correlation between genetic variants and concerned exposure; 2) no connection between genetic variants and confounders that could influence the exposure-outcome relationship; 3) the exclusive pathway that only through exposure do genetic variants affect the outcome. Since no original data were collected, ethical approval and informed consent were not needed for this study.

### 2.2 Data sources and genetic instrumental variants selection

To generate the genetic instrumental variants, the GWAS summary statistics for BP were downloaded online from FinnGen Biobank Documentation of the R12 release, which includes 219 BP cases and 218,066 controls (https://gwas.mrcieu.ac.uk/). The GWAS data for psoriasis vulgaris were extracted from Sakaue et al., which comprises 5072 cases and 478,102 controls (20). Additional quality control steps were performed to select eligible instrumental single nucleotide polymorphisms (SNPs). 1) For Psoriasis vulgaris, we filtered SNPs that met the genome-wide significance criteria of *P*<5×10^−8^; while a lower threshold of *P*<5×10^−6^ for BP since only one SNP was identified at *P*<5×10^−8^ in the BP GWAS summary statistics (21). Thereafter, SNPs were pruned for independence using a clumping procedure with a linkage disequilibrium (LD) threshold of R^2^ > 0.001 in a 10,000 kb window. 2) SNPs were discarded if absent in the outcome GWAS summary statistics. 3) Datasets for exposure and outcome were then harmonized, and palindromic SNPs with an ambiguous strand (i.e., A/T or G/C) were excluded. Additionally, *F* statistics were calculated to evaluate the strength of the selected IVs with the exposure. When the *F* statistic was >10, the likelihood of weak instrument variable bias was low (22). As a result, 13 SNPs associated with psoriasis vulgaris and 11 SNPs linked to BP were selected as the IVs (**Supplementary Table S1**).

### 2.3 Statistical analysis

In this study, the inverse-variance-weighted (IVW) model was used as the primary analysis method, which is most frequently utilized and could provide robust causal evaluations when directional pleiotropy is absent. Additionally, weighted mode, weighted median, MR-Egger regression, and simple mode were employed in extensive analysis to assess the stability of the result. Besides, we presented the effect estimates using odds ratios (ORs) with 95% confidence intervals (CIs) to present the effect estimates. The estimate can be interpreted as the average variation in the outcome per log-odds increase in the genetically predicted risk of the binary exposure (23). In order to lessen the horizontal pleiotropy effect, MR-PRESSO was also used to identify potential outlier instruments (*P*<1), which were then gradually eliminated (24). Heterogeneity tests were performed using IVW and MR-Egger regression, in which heterogeneities were quantified by Cochran Q statistics, and significant heterogeneity was indicated by *P <*0.05 (25). In addition, a leave-one-out analysis was conducted to evaluate the stability of the MR results by eliminating each SNP individually (26). All statistical analyses were conducted using the ‘TwoSampleMR’ and the ‘MR-PRESSO’ packages in software R (version 4.2.1) with statistical significance set at *P <*0.05.

## 3 Results

### 3.1 Causal effects of psoriasis vulgaris on the risk of BP

To investigate the causal effect of psoriasis vulgaris on BP, 13 SNPs were extracted as IVs (**Supplementary Table S1**) and MR-PRESSO detected no outliers. The combined instrument *F* statistic was 168.06, which was much greater than 10, suggesting strong instrument-exposure relationships. The MR estimates demonstrated that genetically predicted psoriasis vulgaris significantly increased the risk of developing BP (IVW: OR=1.263, 95%CI= 1.013–1.575, *P*=0.038, **Table 1**).

**Table 1:** Mendelian randomization estimates from different methods for the relationship between bullous pemphigoid and psoriasis vulgaris.

Outcome	Exposure	nSNPs	Method	OR	95% CI	*P*-Value
Psoriasis vulgaris	Bullous pemphigoid	13	Inverse variance weighted	1.263	1.013-1.575	0.038
			MR Egger	1.315	0.951-1.817	0.126
			Weighted median	1.177	0.889-1.559	0.254
			Simple mode	1.414	0.823-2.429	0.234
			Weighted mode	1.255	0.973-1.618	0.106
Bullous pemphigoid	Psoriasis vulgaris	11	Inverse variance weighted	0.996	0.978-1.015	0.707
			MR Egger	0.994	0.970-1.020	0.675
			Weighted median	0.998	0.974-1.022	0.870
			Simple mode	0.982	0.948-1.018	0.353
			Weighted mode	0.9998	0.969-1.032	0.991

SNP, single nucleotide polymorphism; OR, odds ratios; CI, confidence interval.

**Figure 2:**
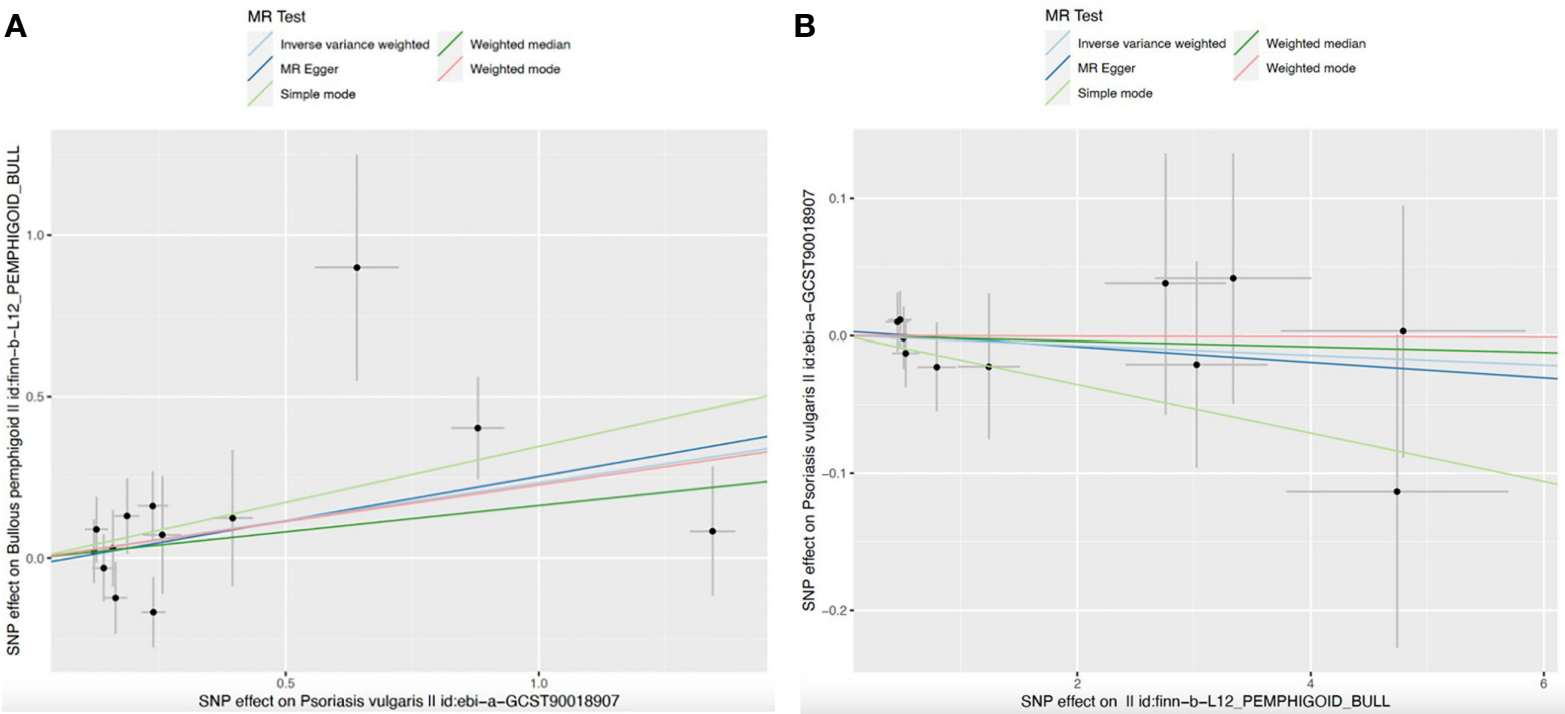
Scatter plots for MR analyses of the causal effect. **(A)** Psoriasis vulgaris-Bullous pemphigoid; **(B)** Bullous pemphigoid–Psoriasis vulgaris. Analyses were conducted using the conventional IVW, weighted median, MR-Egger, weighted mode and simple mode methods. The slope of each line corresponding to the estimated MR effect per method.

For the sensitivity analyses, the weighted mode (OR=1.255, 95%CI: 0.973-1.618, *P*=0.106), MR Egger (OR=1.315, 95%CI: 0.951-1.817, *P*=0.126), simple mode (OR=1.414, 95%CI: 0.823-2.429, *P*=0.234), and weighted median method (OR=1.177, 95%CI: 0.889-1.559, *P*=0.254), derived directionally consistent relationship between the genetically predicted log odds of psoriasis vulgaris and the risk of developing BP. The scatter plots of the SNP impact sizes of psoriasis vulgaris on IBD by different MR methods are displayed in **Figure 2A**. Furthermore, the MR-Egger analysis did not reveal horizontal pleiotropy (intercept =-0.0203, *P* =0.7380) (**Table 2**) and there was no heterogeneity among these IVs according to Cochran’s Q report (*P*>0.05). The funnel plot (**Supplementary Figure S1A**) showed an overall symmetric inverted funnel shape. According to the leave-one-out plots (**Supplementary Figure S2A**), there were no potentially significant SNPs driving the causative link; instead, the consequences were not driven by any particular extreme SNPs but rather by an overall interrelated pattern of the correlations.

**Table 2:** Sensitivity analyses of MR.

Exposure	Outcome	Heterogeneity test			MR-egger pleiotropy test		
		Q	Df	*P*-value	Intercept	SE	P-value
Psoriasis vulgaris	Bullous pemphigoid	16.227	12	0.181	-0.020	0.059	0.738
Bullous pemphigoid	Psoriasis vulgaris	2.764	10	0.986	0.003	0.014	0.821

SE, Standard error.

### 3.2 Causal effects of BP on the risk of psoriasis vulgaris

Through reverse MR analysis, 13 SNPs were identified as instrumental variables (IVs) for BP following LD operation (R^2^ > 0.001 in a 10,000 kb window) at the GWAS level of *P <*5×10^−6^. After removing all outlier SNPs detected by the MR-PRESSO approach, 11 of them (*F*=25.518) were included in the final analyses to examine the causal effect of BP on psoriasis vulgaris. The *F* statistic much higher than 10 indicates a substantial correlation between the instruments and exposure. **Supplementary Table S2** provides comprehensive details regarding the IVs used in bidirectional MR studies.

Our results indicated that genetically predicted BP had no significant effect on psoriasis vulgaris for IVW (OR=0.996, 95%CI=0.978-1.015, *P*=0.707), MR-egger (OR=0.994, 95%CI=0.970-1.020, *P*=0.675), weighted median (OR=0.998, 95%CI=0.974-1.022, *P*=0.870), simple mode (OR=0.982, 95%CI=0.948-1.018, *P*=0.3528), and weighted mode (OR=0.9998, 95%CI=0.969-1.032, *P*=0.991) (**Table 1**; **Figure 2B**). Additionally, scatter plots of the causal link between BP and psoriasis vulgaris were shown in **Figure 2B**. All sensitivity studies revealed that the inconspicuous correlation remained impartial. Furthermore, no potential horizontal pleiotropy was shown by the MR-Egger regression (all *P*_intercept_ > 0.1). The Cochran’s Q value (*P* for Q >0.1) also did not imply heterogeneity. The funnel plots are shown in **Supplementary Figure S1B**. No obvious anomalies were seen in the leave-one-out analysis (**Supplementary Figure S2B**).

## 4 Discussion

The present study, to the best of our knowledge, was the first MR analysis study to evaluate the bidirectional causal relationships between psoriasis vulgaris and BP. It’s interesting to note that psoriasis vulgaris was significantly correlated with a higher risk of BP, employing genetic instruments from the top most accessible GWAS data. However, no evidence was discovered by reverse MR to support the role of BP in the risk of psoriasis vulgaris.

In recent years, several cases and clinical studies have demonstrated the potential relationship between psoriasis vulgaris and BP (8, 14, 15). In one study that examined 145 cases of coexisting autoimmune bullous diseases (AIBDs) and psoriasis, the most prevalent AIBD in patients with psoriasis was BP (63.4%) and the most prevalent type of psoriasis was psoriasis vulgaris (14), indicating the potential association between psoriasis vulgaris and BP. Another study also demonstrated that patients with BP had a higher prevalence of psoriasis than those without BP (5.2% vs. 1.2%; OR, 4.4; 95% CI, 2.2-8.9; P<0.0001) and psoriasis vulgaris was the most common type (8). Furthermore, psoriasis was developed before BP in 93.3% of the patients who had both conditions (8). According to a literature review containing 40 case reports (7), psoriasis predated BP in most of the instances, with an average interval of 20 years (range: 1–60 years). The occurrence of psoriasis prior to BP in most cases indicated the potential impact of psoriasis on developing BP. However, since the results of abovementioned observational studies might be influenced by uncontrolled residual confounding factors such as comorbidities, lifestyle, and socioeconomic levels (8, 27, 28), the relationship between psoriasis and BP as well as the direction of the relationship remained undetermined. Thus, in the present study, we employed an MR approach, which is free of common confounding biases existing in observational studies (29), to determine the causal relationship between psoriasis vulgaris and BP. Overall, the result of our MR analysis was consistent with the abovementioned observational studies, and further demonstrated the causal effects of psoriasis vulgaris on BP.

However, the risk of psoriasis after BP remains unclear. A survey of 104,669 Japanese patients with psoriasis revealed that treating bullous diseases with corticosteroids was likely to trigger an immediate development of pustular psoriasis. In contrast, evidence is scarce for psoriasis vulgaris developing after BP in previous research (30). Our MR analyses did not detect a pronounced role of BP in the risk of psoriasis vulgaris either. Combined with previous research, we revealed an interesting phenomenon that psoriasis vulgaris causally increases the subsequent BP risk, yet a significant effect of BP on psoriasis vulgaris development was not found. The reason for the phenomenon remained unclear.

Previous basic research initially investigated the coexisting mechanism of BP and psoriasis. Multiple investigations have shown similar dysregulated cytokine expression and inflammatory responses in the immune systems of psoriasis and bullous pemphigoid patients (27, 31, 32). For instance, IL-1 was found necessary for the development of psoriatic lesions such as epidermal proliferation and differentiation (31), and the level of IL-1b was found to be higher in blister fluid than in BP serum (33). The shared cytokines may play a part in the coexistence of psoriasis and AIBD. Furthermore, the keratinocytes present in both psoriasis and BP can produce neutrophil chemokines, such as (IL)-8, resulting in a common histological feature of neutrophil infiltration, which further leads to the releasing of various metalloproteinases, including disintegrant and metalloproteinases ADAM9, ADAM10, and ADAM17. These enzymes contribute to extensive degradation of matrix proteins and subsequent exposure of self-epitopes at the dermal-epidermal junction (27). In addition, prior research has demonstrated that both involved and uninvolved psoriatic lesions in patients with psoriasis vulgaris exhibit disruptions to laminin 1 and laminin a1 inside the basement membrane zone (BMZ) (14, 34). The study hypothesized that the antigenicity of basement membranes in patients with psoriasis began to decrease at a younger age, which promotes the generation of antibasement membrane autoantibodies and the development of BP earlier in life (14). These partially explained our findings that psoriasis vulgaris causally increases the risk of BP development, possibly due to the neutrophil infiltration or other systemic inflammation of psoriasis vulgaris that may cause exposure of self-epitopes at the dermal-epidermal junction and further induce BP.

However, despite the similar cytokines found in the pathogenesis of psoriasis and BP, some cytokines of the two diseases were quite different, indicating different pathogenesis. While the IL-23/T-helper (Th)17 inflammatory pathway, which involves tumor necrosis factor (TNF)-alpha, IL-23, and IL-17, is critically involved in psoriasis pathogenesis (35, 36), the Th-2 pathway, primarily involving IL-4, IL-5, IL-6, IL-10, IL-8, IL-18, and IL-31, has been identified as the essential driver of BP antibody production (37–40). The complexity may partially explain why BP cannot increase psoriasis vulgaris in turn. Psoriasis with BP may offer an intriguing representation of the intricacy of the cutaneous inflammatory networks (41).

Partially due to the complexities of cutaneous inflammatory networks, some of the treatments for psoriasis vulgaris or BP are quite selective (42). On one hand, several anecdotal cases of autoimmune bullous diseases were reported to be caused by biologics targeting important cytokines in psoriasis (43–45). Besides, while UV radiation has shown efficacy in treating psoriasis, they were found to exacerbate or induce BP (46). On the other hand, although dupilumab, a dual inhibitor of IL-4 and IL-13 signaling, has been used successfully in BP, it is generally known to cause psoriasis (47). The contradictory nature of above treatment suggested the complex relationship between the two diseases. Thus, despite the causal effects of psoriasis vulgaris on BP and the insignificant effect of BP on psoriasis vulgaris found in this study, psoriasis should be treated with caution to prevent the onset of BP, and vice versa. Fortunately, some previous findings suggested that tofacitinib could be a safe and useful treatment for people with combined psoriasis and BP, which shed light on the treatment guidance for both comorbid disorders (48, 49). To realize precise prevention and early diagnosis, the exact path mechanism underlying the causal relationship is warranted to be investigated deeply.

This MR study investigated the causal relationship between psoriasis vulgaris and BP and possessed several strengths over previous studies. First, due to the MR study design, this study is free of many biases inherent in observational studies, such as confounding, reverse causality, and so on (29). Second, since the primary IVW method is prone to horizontal pleiotropy, additional robust MR analytical methods were applied in the sensitivity analysis. Furthermore, outlier SNPs identified in the MR-PRESSO outlier test were excluded in the final analysis, which reduced the possibility of horizontal pleiotropy and enhanced the reliability of our results (50).

Nevertheless, the present study was not free from limitations. First of all, although various methods were utilized in the sensitivity analyses to enhance the robustness of results, the difference in the significance of the results among different MR methods made the conclusion obscure, especially for the effect of psoriasis vulgaris on BP. However, the OR results by all the employed methods were directionally consistent and the OR values were strong, indicating the existence of the causal effects of psoriasis on BP (**Table 1**). Secondly, the data source is limited in two ways: 1) the sample size of BP is relatively small; and 2) only psoriasis vulgaris in relation to BP was investigated, while other forms of psoriasis remain unclear. So, further GWAS data and studies are needed. Thirdly, the GWAS datasets employed in our research were based on European. So our results may not be fully representative of the whole population. Moreover, it is worth noting that population stratification, a common confounding factor, may bring about false–positive genetic associations, which result in biased IVs for MR (21, 50). Therefore, a larger multi-racial GWAS will boost the efficiency of future MR studies to clarify the actual relationship between psoriasis vulgaris and BP, and we should carefully utilize our conclusion in racially and ethnically diverse populations. Fourthly, the potential pleiotropy effect in our study cannot be cleared off, although the MR-Egger intercept in our study indicated little horizontal pleiotropism and we took steps to identify and exclude outlier variants (51). Lastly, in the majority of cases, genetic variation may only account for a tiny percentage of exposures or traits (52, 53). The listed limitations suggested that the risk of developing psoriasis vulgaris in patients with BP might remain to be confirmed, and vice versa. Although we found no evidence for the causal effects of BP on psoriasis vulgaris, further studies are needed to finally clarify the subject.

## 5 Conclusion

Based on our bidirectional two-sample MR analysis, genetically predicted psoriasis vulgaris is causally associated with an increased risk of developing BP. Foremost, our research serves as a reminder to clinicians that in daily clinical practices, heightened attention should be given to the emergence of symptoms such as bullous tense blisters, urticarial plaques, or erythema in patients with psoriasis vulgaris, enabling early diagnosis and timely treatment of BP. The biological mechanisms behind this causality are complex and needed to be investigated in further studies, in order to decrease the occurrence of new-onset BP in psoriasis vulgaris patients as well as determine the investment in screening concomitant conditions in both directions.

## Data availability statement

The original contributions presented in the study are included in the article/**Supplementary Material**. Further inquiries can be directed to the corresponding author.

## Author contributions

AZ: Writing – review & editing, Writing – original draft, Software, Methodology, Investigation, Formal analysis, Data curation, Conceptualization. ZY: Writing – review & editing, Supervision, Software, Methodology, Investigation, Formal analysis, Data curation, Conceptualization. TH: Writing – review & editing, Project administration, Methodology, Formal analysis. MW: Writing – review & editing, Supervision, Resources, Project administration, Conceptualization.
