# Supplementary material for: Causal association between psoriasis vulgaris and bullous pemphigoid: a two-sample bidirectional Mendelian randomization study
Source: Front Immunol. 2024 Mar 13;15:1365118. doi: 10.3389/fimmu.2024.1365118 (PMC10965669; doi:10.3389/fimmu.2024.1365118)
Supplement: Supplementary file 1 [file DataSheet_1.docx]

# Supplementary tables and figures

**Table S1**. Detailed information of LD-independent SNPs associated with psoriasis vulgaris that were selected as instrument variables

| No. | SNP | Effect allele | Other allele | Beta exposure | SE Exposure | *P*_value_exposure | Effective_sample_size | Chromosome | Position |
| --- | --- | --- | --- | --- | --- | --- | --- | --- | --- |
| 1 | rs10040411 | T | C | -0.2372 | 0.0297 | 1.45E-15 | 483174 | 5 | 158814116 |
| 2 | rs1177203 | C | G | -0.1211 | 0.0205 | 3.77E-09 | 483174 | 2 | 61074686 |
| 3 | rs12188300 | T | A | 0.3952 | 0.0397 | 2.57E-23 | 483174 | 5 | 158829527 |
| 4 | rs147748716 | T | C | 0.6407 | 0.0822 | 6.56E-15 | 483174 | 6 | 31321240 |
| 5 | rs2249937 | G | T | -0.158 | 0.0233 | 1.18E-11 | 483174 | 6 | 159515309 |
| 6 | rs2312786 | A | G | 0.1403 | 0.0229 | 9.61E-10 | 483174 | 3 | 101633360 |
| 7 | rs2735008 | A | G | -0.1863 | 0.0246 | 3.82E-14 | 483174 | 6 | 29808013 |
| 8 | rs28752856 | G | C | 0.8795 | 0.0512 | 3.90E-66 | 483174 | 6 | 31298418 |
| 9 | rs28894993 | T | G | 1.3435 | 0.0437 | 1.00E-200 | 483174 | 6 | 31289714 |
| 10 | rs34725611 | G | A | -0.1636 | 0.0225 | 3.80E-13 | 483174 | 19 | 10477067 |
| 11 | rs6933987 | T | C | 0.1258 | 0.0223 | 1.78E-08 | 483174 | 6 | 138239434 |
| 12 | rs9264277 | C | T | 0.2385 | 0.0233 | 1.34E-24 | 483174 | 6 | 31224667 |
| 13 | rs9481169 | T | G | 0.256 | 0.0373 | 6.58E-12 | 483174 | 6 | 111929862 |

| No. | SNP | Effect allele | Other allele | Beta exposure | SE Exposure | P_value_exposure | Effective_sample_size | Chromosome | Position |
| --- | --- | --- | --- | --- | --- | --- | --- | --- | --- |
| 1 | rs10483424 | A | G | 3.3364 | 0.6673 | 5.73E-07 | 218285 | 14 | 33591131 |
| 2 | rs11205458 | G | C | 0.4553 | 0.0995 | 4.71E-06 | 218285 | 1 | 48695658 |
| 3 | rs144298683 | T | C | 2.7543 | 0.5178 | 1.05E-07 | 218285 | 3 | 88344440 |
| 4 | rs145834662 | A | T | 4.7943 | 1.0489 | 4.86E-06 | 218285 | 2 | 85296873 |
| 5 | rs147633772 | T | A | 1.3754 | 0.2151 | 1.60E-10 | 218285 | 6 | 32613983 |
| 6 | rs200148 | A | G | -0.479 | 0.0989 | 1.27E-06 | 218285 | 6 | 143345696 |
| 7 | rs3094169 | C | T | -0.5736 | 0.1229 | 3.04E-06 | 218285 | 6 | 29829986 |
| 8 | rs35604167 | T | G | 0.7932 | 0.1627 | 1.09E-06 | 218285 | 4 | 169382451 |
| 9 | rs60559879 | A | G | 0.5073 | 0.11 | 3.99E-06 | 218285 | 17 | 35192062 |
| 10 | rs75308030 | T | G | 4.7413 | 0.9513 | 6.23E-07 | 218285 | 10 | 26019862 |
| 11 | rs76965871 | A | G | 3.0219 | 0.6069 | 6.37E-07 | 218285 | 1 | 175729422 |
| 12 | rs79334883 | G | A | 1.2389 | 0.2667 | 3.38E-06 | 218285 | 4 | 86211322 |
| 13 | rs9996810 | C | T | 0.5263 | 0.1148 | 4.52E-06 | 218285 | 4 | 62468670 |

**Table S2**. Detailed information of LD-independent SNPs associated with bullous pemphigoid that were selected as instrument variables

**Figure S1.**

| A  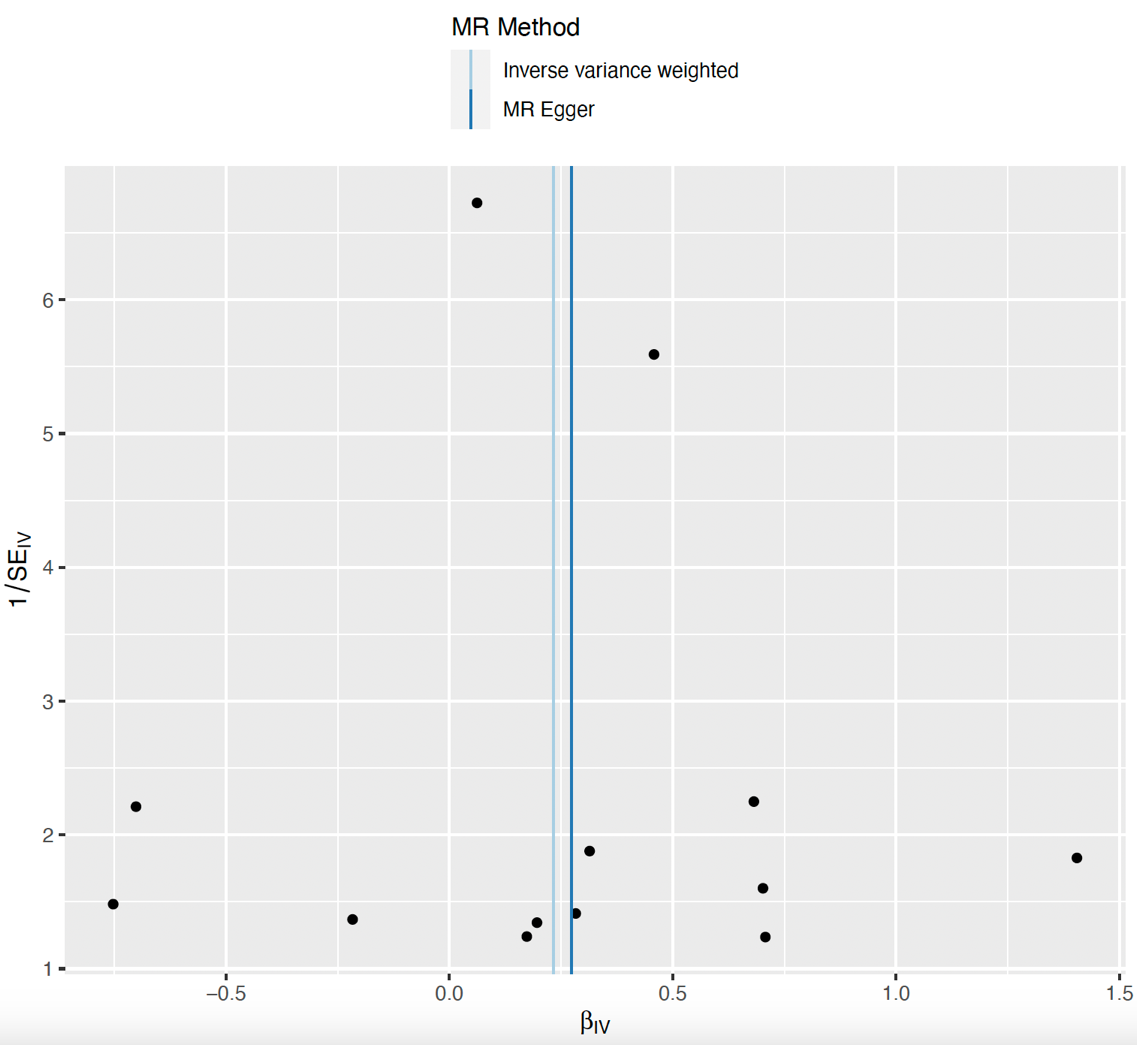 | B  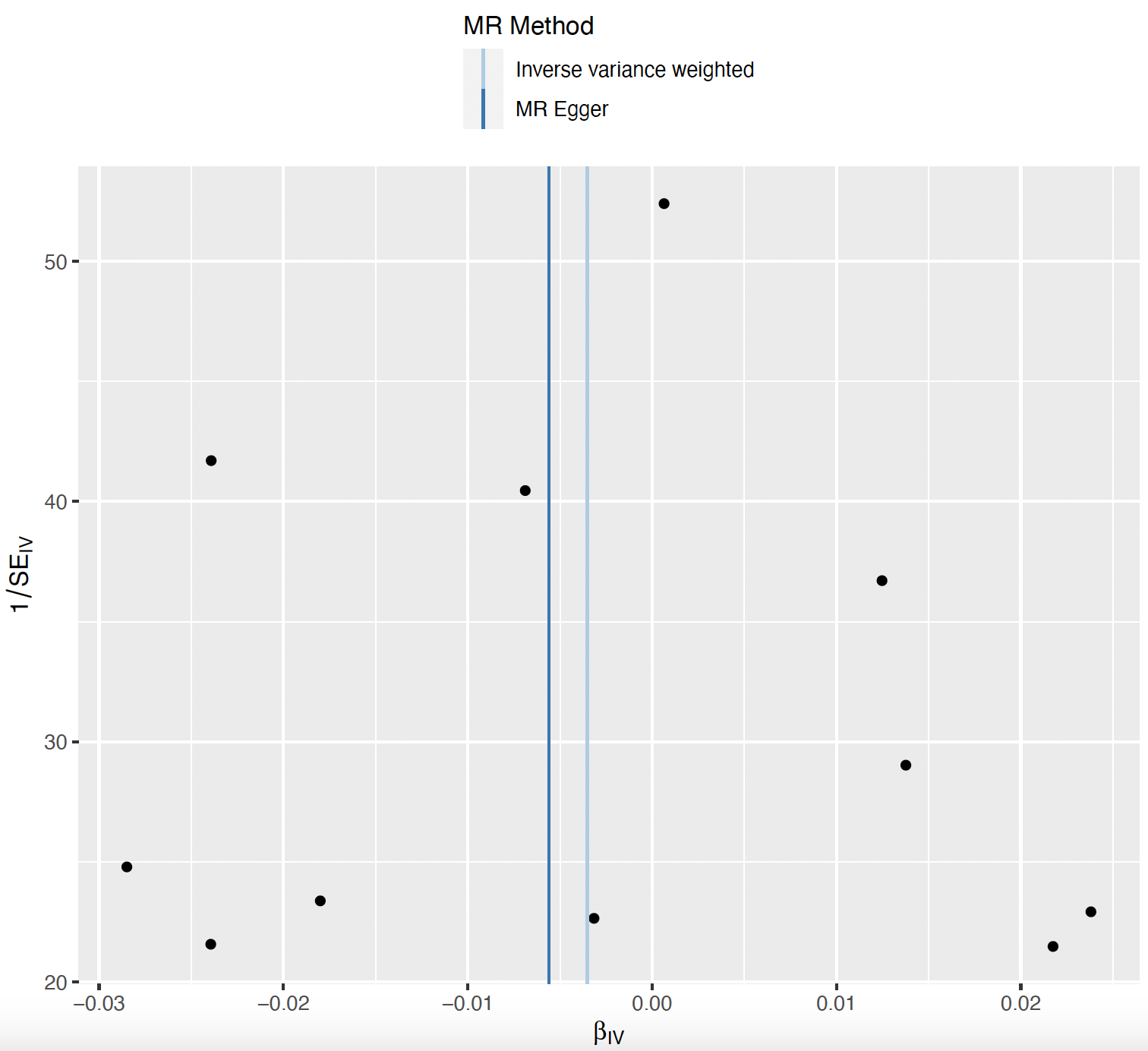 |
| --- | --- |

**Figure S1**. Funnel plots for MR directional horizontal pleiotropy analyses for the causal effects of psoriasis on bullous pemphigoid(A), and bullous pemphigoid on psoriasis (B).

**Figure S2.**

| **A**  **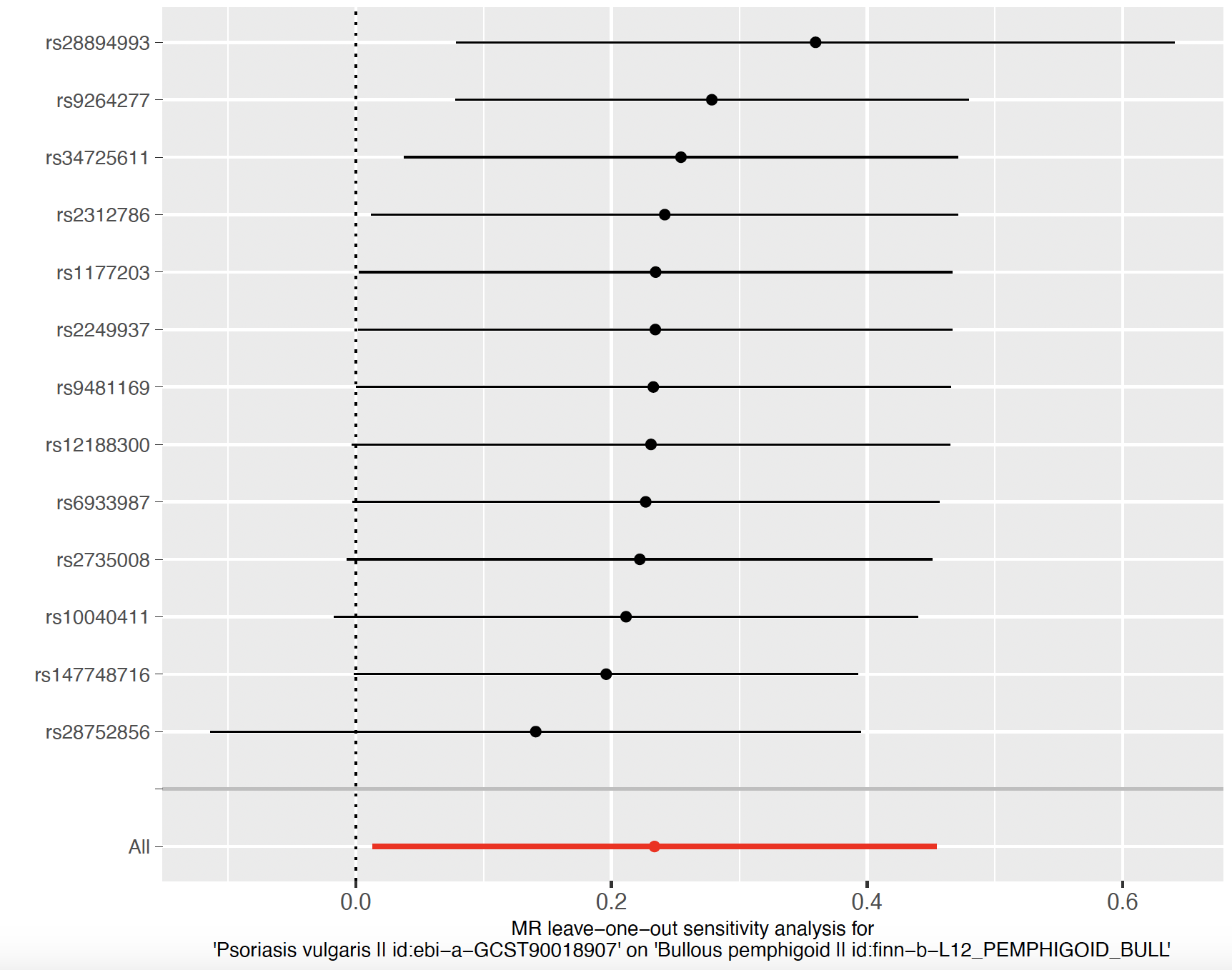** | **B**  **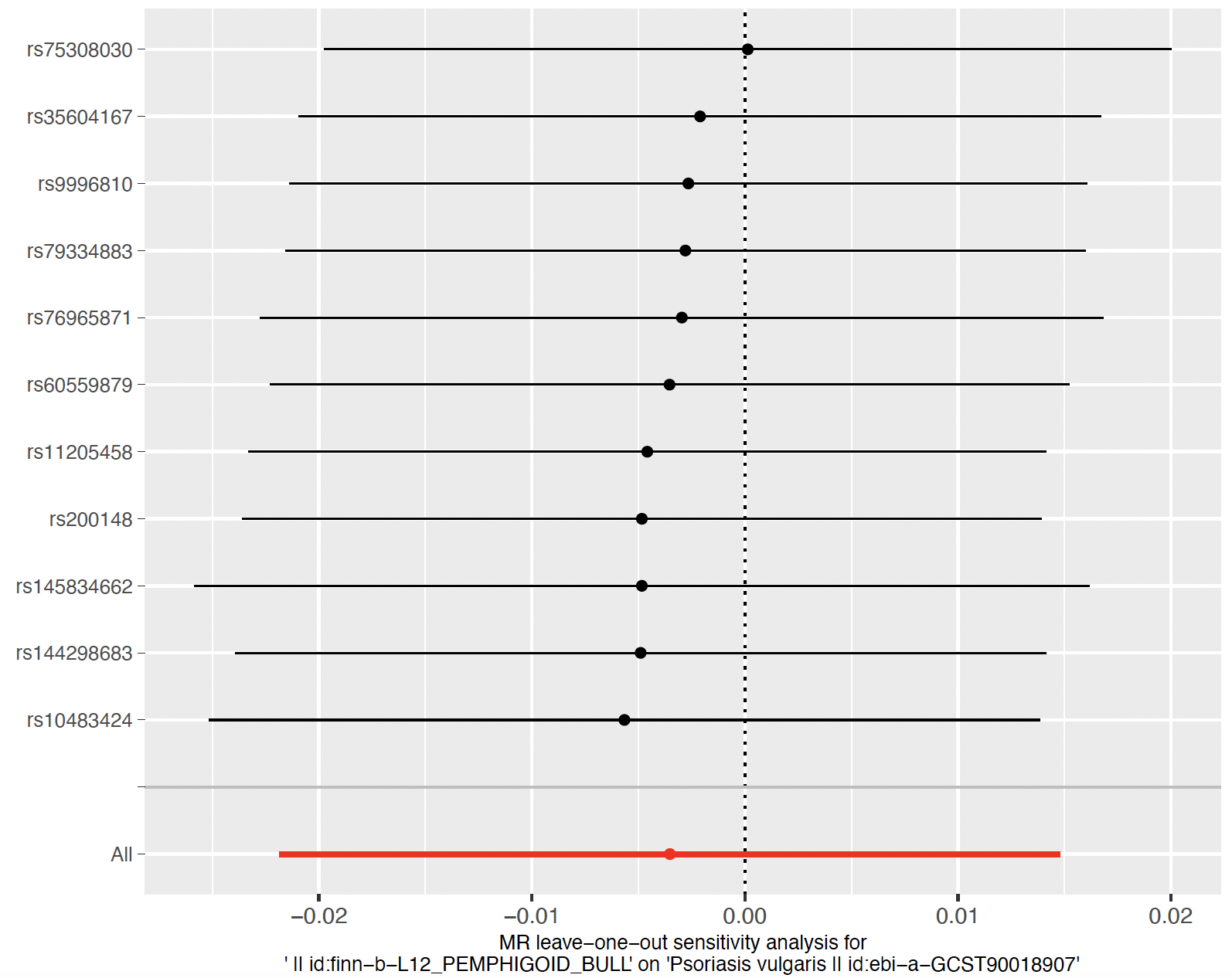** |
| --- | --- |

**Figure S2**. MR leave-one-out sensitivity analysis for psoriasis on bullous pemphigoid(A), and bullous pemphigoid on psoriasis (B).
